# The Cape Hospital Ordinance

**Published:** 1913-01-18

**Authors:** 


### The Hospital

The Workers' Newspaper of Administrative Medicine and Institutional Life, Administration, National Insurance and Health.

No. 1385, Vol. LIII. SATURDAY, JANUARY 18, 1913. PRICE ONE PENNY.

### THE CAPE HOSPITAL ORDINANCE.

In a coming issue we propose to publish a *résumé* of Dr. Thornton's able and interesting Report on the condition of the hospitals in the Cape Province of the Union of South Africa, together with a summary of the suggested reforms which have now been embodied in a special Hospital Ordinance. The interest of the Report and the Ordinance is the greater because both deal with certain questions which at the present moment are of importance to the hospital world in this country. The one is the question of hospital abuse and the manner in which it may be counteracted; the other is the even more vital problem of hospital finance in view of the increasing demand for hospital treatment of a class of the community that can afford to pay for hospital treatment. In view of these facts Dr. Thornton's Report deserves to be studied by every hospital worker.

A new country, where the voluntary principle has never been held so strongly as it has been adhered to in England, presents, so far as hospital questions are concerned, wide differences from our own; where the population is small, and especially where there is a large class of indigent natives, Government aid in support of the hospitals is not merely excusable but is indispensable. That is the reason for the Ordinances in the West Indian Islands and in New Zealand, where the £ for £ principle has been recognised as an equitable basis for a settlement safeguarding, on the one hand, the Government's right to inspect the hospitals, and, on the other, the right of the institutions to be independent bodies democratically managed by their subscribers. At the Cape there has hitherto been no uniformity either in the management of hospitals or in the apportioning of the State grants in aid. The result has been the introduction of abuses both in the administration and in what may be called the essentials of hospital politics. Dr. Thornton's Report shows instances of these abuses; he refers to the overlapping, to the over-bedding of hospitals, and to the extravagance and waste which are direct results of both.

The new Ordinance, which has received the approval of the Provincial Council after a somewhat stormy passage in debate, establishes a uniform system for the whole of the Cape Province in regard to hospital management. It provides for the democratic control of the hospitals by means of boards whose members are chosen from among all those who are directly concerned in the working of the institutions; and it is specially to be noted that adequate provision is made for the representation of the medical staff on these boards. It provides further for a proper classification of patients into two categories: paying patients, housed in special pay wards, who have the right of calling in their own medical attendant if they elect to do so, and poor or free patients who rank as do the ordinary inmates of our voluntary hospitals. Special clauses enable the hospital authorities to safeguard themselves against abuse by empowering them to institute inquiries from each applying patient, who is bound by law to give them all information regarding his means, false information being punishable, and to regain the cost of treatment from such patients as can afford to pay. Such a proviso is an undoubted advance upon the system of voluntary almoners we possess, but it is one of the legislative benefits that only a new community could confer upon its charities, and there is but little hope that we shall ever meet its like in any English enactment.

More important and more interesting, however, than these details of administration are the provisions with regard to finance and central control. Under the Ordinance every hospital may claim from the Government certain grants in aid apportioned on practically three main scales. First, as regards voluntary contributions, the Government pays the hospital twenty-five shillings for every twenty shillings it raises in subscriptions. This scale necessarily means an inducement to the energetic hospital secretary to increase the number of his annual subscribers; it is in fact the only basis on which, under a system of State aid, the voluntary principle can be effectively encouraged. Secondly, the Government pays, on the £ for £ principle, equal sums to those collected from paying patients or bequeathed for special purposes, provided that no single grant made in respect of bequests exceeds £500. Thirdly, under certain conditions, when it has been shown that the hospital board has made reasonable efforts to wipe out its annual deficit, the

Government may grant a sum not exceeding half the amount of such deficit remaining, the other half being paid by the local authority from rates collected on the immovable property within the hospital area. These provisions are not new; they are taken, in substance, from the New Zealand and Australian Acts, where the principle of allocation on the basis of the ordinary revenue from all sources has worked extremely well. As in these Dominions, so possibly at the Cape, the immediate result of the Ordinance will be a falling-off in the ordinary revenues of hospitals. State aid sounds grand to subscribers of an institution that has always been appealing for funds; the notion that there is the Government to fall back upon for liquidating a deficit appeals to those who have had experience of the difficulties in collecting money. But it is necessary to note that the Ordinance safeguards certain essentials of the voluntary principle. If a hospital's voluntary revenue falls its Government grant will correspondingly decrease, and the time will come when it will cease to be an efficient institution. Only by promoting a vigorous and healthy hospital spirit in its district can a hospital hope to flourish when it receives Government or municipal assistance on the basis on which the Cape hospitals will receive aid under the Ordinance.

The question of central control has been carefully, and we think wisely, handled in the Ordinance. The Government exercises direct control over the finances, in so far as the institutions have to submit their estimates to the department which has the right to veto expenditure. For the rest, while the Government reserves the right of periodically inspecting the hospitals, it does not in the least interfere in the management or administration. All that it does is to give judicious advice, and when we read Dr. Thornton's Report we must confess that it seems that the Cape hospitals are often in need of some expert mentor. The principle of democratic control, which is one of the features of our own system, is therefore safeguarded to a large extent, while every inducement is held out to the public to become actively interested in hospitals and to manage them in much the same manner as our own institutions are administered. In this respect it seems to us that the South African Act is an improvement upon that of New Zealand, where the Government has wider powers of control and greater opportunities of interference.

